# Medial temporal lobe-dependent repetition suppression and enhancement due to implicit vs. explicit processing of individual repeated search displays

**DOI:** 10.3389/fnhum.2012.00272

**Published:** 2012-10-04

**Authors:** Thomas Geyer, Florian Baumgartner, Hermann J. Müller, Stefan Pollmann

**Affiliations:** ^1^Unit of General and Experimental Psychology, Department of Psychology, Ludwig-Maximilians-Universität MünchenMünchen, Germany; ^2^Institut für Psychologie II, Otto-von-Guericke-Universität MagdeburgMagdeburg, Germany; ^3^School of Psychology, Birkbeck College, University of LondonLondon, UK; ^4^Center for Brain and Behavioral Sciences, Otto-von-Guericke-Universität MagdeburgMagdeburg, Germany

**Keywords:** visual search, contextual cueing, medial temporal lobe, awareness

## Abstract

Using visual search, functional magnetic resonance imaging (fMRI) and patient studies have demonstrated that medial temporal lobe (MTL) structures differentiate repeated from novel displays—even when observers are unaware of display repetitions. This suggests a role for MTL in both explicit and, importantly, implicit learning of repeated sensory information (Greene et al., 2007). However, recent behavioral studies suggest, by examining visual search and recognition performance concurrently, that observers have explicit knowledge of at least some of the repeated displays (Geyer et al., 2010). The aim of the present fMRI study was thus to contribute new evidence regarding the contribution of MTL structures to explicit vs. implicit learning in visual search. It was found that MTL activation was increased for explicit and, respectively, decreased for implicit relative to baseline displays. These activation differences were most pronounced in left anterior parahippocampal cortex (aPHC), especially when observers were highly trained on the repeated displays. The data are taken to suggest that explicit and implicit memory processes are linked within MTL structures, but expressed via functionally separable mechanisms (repetition-enhancement vs. -suppression). They further show that repetition effects in visual search would have to be investigated at the display level.

## Introduction

One of the key paradigms for studying human perception and attention is visual search. In this paradigm, observers are presented with an array of stimuli, one of which is the to-be-detected and/or -discriminated target item and the others are non-target (distractor) items. Previous studies have revealed a number of mechanisms that can effectively guide search for the target (cf. Wolfe, 1998). This article is concerned with one of these mechanisms: “contextual cueing” (e.g., Chun and Jiang, 1998). Contextual cueing refers to the fact that when a target is repeatedly encountered, over the course of an experiment, at an invariant position within the same distractor arrangement (context), target detection is expedited relative to displays with non-repeated, random distractor arrangements—even though observers are typically unable to consciously recognize such repeated distractor contexts. These findings have been taken to mean that contextual cueing is supported by an implicit memory system which guides focal attention more rapidly towards the target location (though there may also be some contribution of contextual cueing to response selection—see Kunar et al., 2007). The present functional magnetic resonance imaging (fMRI) study was designed to address three open issues as to the memory mechanisms underlying contextual cueing—by pursuing a novel approach, namely, that of assessing contextual cueing and recognition performance in visual search concurrently (see e.g., Smyth and Shanks, 2008); the issues are: (1) whether medial temporal lobe (MTL) structures support also implicit, in addition to explicit, learning of repeated target-distractor arrangements in visual search [the position advocated by, e.g., Greene et al. (2007)] or whether MTL-dependent learning is contingent on awareness of repeated displays [the position advocated by, e.g., Squire (1992)]; (2) how hemodynamic activity in response to displays of which observers do or do not have explicit knowledge has to be characterized (repetition-enhancement vs. -suppression); and (3) at which processing stage (learning vs. expression of learning) any performance differences between “explicit” and “implicit” displays may become manifest.

The contextual-cueing paradigm was first introduced by Chun and Jiang (1998). They had observers search for a target letter “T,” oriented 90° from the vertical to either the left or the right, presented within an array of heterogeneously oriented “L” distractors; observers had to discriminate the target T's orientation (left vs. right) as quickly and as accurately as possible (a target was present on each trial). There were two conditions: first, a repeated condition, in which the target appeared at a number of pre-defined locations within invariant distractor configurations. Second, a non-repeated condition, in which targets were presented amongst distractors whose locations were chosen randomly on each trial in an experimental block. Importantly, in the latter condition, the target also appeared at a limited number of pre-defined locations (which were, of course, different from those in the repeated condition) to equate target location repetition effects between the conditions. It was found that targets appearing in invariant contexts were detected and discriminated more rapidly than targets in randomly variable contexts—an effect which has been referred to as “contextual cueing.” Further, when observers were asked about repeated displays in an explicit-knowledge test—for instance, when they had to predict or generate the location of a missing target in a repeated display (e.g., Chun and Phelps, 1999; Chun and Jiang, 2003) or perform a forced-choice recognition test (e.g., Chun and Jiang, 1998; Manns and Squire, 2001)—their performance was effectively at chance level. Findings along these lines led Chun and Jiang (1998) to propose that the memory underlying contextual cueing is implicit. However, it is important to note that these studies used only very small numbers of recognition trials (typically 24 recognition trials; e.g., Chun and Jiang, 2003; Greene et al., 2007). By contrast, memory-based influences on reaction time (RT) performance have been assessed presenting hundreds of search trials (typically 576 search trials; e.g., Chun and Jiang, 1998). Accordingly, explicit memory effects would have to be very large to be disclosed statistically with these small numbers of recognition trials.

This “power problem” of recognition tests was addressed in recent studies (e.g., Smyth and Shanks, 2008; Geyer et al., 2010). Geyer et al. (2010) administered a forced-choice recognition test after each of their 32 search blocks (yielding a total of 512 recognition trials) and analyzed recognition performance (i.e., hit rates) separately for each of the repeated displays. Interestingly, it was found that participants were well able to correctly identify a limited number of about 4 (out of a total of 12) repeated displays. Note that in Geyer et al. (2010), a repeated display was classified as “explicit” only if its associated hit rate (uncorrected) was larger than 0.75: bootstrap simulations had shown this value to correspond to an associated level of confidence of 99%, that is, a relatively conservative criterion for determining “explicit” displays.

The finding that observers acquire explicit knowledge of at least some of the repeated displays bears directly on a recent fMRI study of the contextual-cueing effect (Greene et al., 2007). This study revealed differential activity in the hippocampus (HC) between repeated and non-repeated displays, specifically: HC activation was lower for repeated than for non-repeated displays. Based on this finding, and supported by patient studies (e.g., Chun and Phelps, 1999), Greene et al. (2007) concluded “… that the hippocampus plays a potentially important role in the implicit contextual cueing task … ” (p. 552).

These findings, however, were only partially replicated in further, fMRI and patient, studies. For example, Preston and Gabrieli (2008) reported activation changes associated with the size of the contextual cueing effect (positive correlation) in the left entorhinal and perirhinal cortex, but not in HC. Furthermore, in a patient study, Manns and Squire (2001) compared widespread lesions of the MTL, as in the study of Chun and Phelps (1999), with more focal HC lesions and replicated a contextual cueing effect in the latter (in Manns and Squire's terms “H+”) group, but not former (“MTL+”) group. Thus, although some patient and imaging studies (Chun and Phelps, 1999; Greene et al., 2007) suggested hippocampal involvement in contextual cueing, subsequent reports (Manns and Squire, 2001; Preston and Gabrieli, 2008) pointed rather to a role of MTL structures other than the HC as contributing to the cueing effect. Given this, it is an open issue why some studies found HC-dependent contextual cueing (Greene et al., 2007), while others failed to demonstrate a role of the HC in the context-based guidance of visual attention (e.g., Preston and Gabrieli, 2008).

One factor contributing to this apparent discrepancy might be observers' explicit knowledge about repeated displays. For example, when observers are aware of display repetitions, as the findings of, say, Smyth and Shanks (2008) suggest, blood oxygen level-dependent (BOLD) responses elicited by repeated displays might also be modulated by this knowledge. Given this, findings from studies comparing mean HC activity between repeated and non-repeated displays (Greene et al., 2007) would be open to alternative interpretations—for example, that the HC is involved in explicit rather than, or in addition to, implicit learning of repeated search displays (Manns and Squire, 2001; Preston and Gabrieli, 2008). For example, Preston and Gabrieli (2008) found that HC, in addition to parahippocampal, activity was discrepant for recognized relative to unrecognized distractor contexts (see below).

Note, though, that while the above reports are suggestive of a distinction between explicit memory processes in the HC and implicit processes in MTL structures outside the HC, such a dual-view perspective might be too simple in view of recent reports that MTL areas, in particular, the posterior portion of the parahippocampal cortex (PHC), are involved in the explicit learning of spatial contexts (Aminoff et al., 2007). In this regard, Henke (2010) has recently argued that consciousness provides an inappropriate criterion for distinguishing between basic forms of memory such as episodic or procedural memory, priming, etc. Instead, she proposed that memory phenomena ought to be classified on the basis of task requirements, such as the number of learning trials, or cognitive complexity, or the required flexibility of the resulting memory representation. Henke's (2010) proposal is interesting because it assumes that explicit and implicit memory effects are supported by a common memory structure, namely: PHC—which is, on her account, responsible for the rapid encoding of “unitized” items or, in the present context, the learning of repeated distractor arrangements (Henke, personal communication, June 2011). This would imply that PHC in particular is involved in both the explicit and implicit learning of repeated search displays.

On this background, one aim of the present study was to examine whether MTL activity is modulated by knowledge about display repetitions. To examine this, observers performed a learning session (comprising of some 600 search and 150 recognition trials) which was either preceded or followed by an fMRI session (comprising of some 200 search trials only). The purpose of the learning session was to enable configural learning of repeated displays as well as to assess explicit knowledge of the repeated displays. The purpose of the fMRI sessions was to examine MTL activity associated with the processing of displays of which observers did or did not have explicit knowledge—with the explicit-implicit distinction being made with regard to observers' recognition performance obtained on the recognition trials administered in the learning session. Based on prior studies (e.g., Aminoff et al., 2007; Preston and Gabrieli, 2008; Henke, 2010), analyses of MTL activity were limited to three regions-of-interest (ROIs): (1) HC, (2) anterior, and (3) posterior PHC (aPHC and pPHC, respectively).

To our knowledge, only two recent fMRI-studies have addressed the issue of explicit learning in contextual cueing. One study found that both hippocampal and parahippocampal activity differed between explicit and implicit displays (Preston and Gabrieli, 2008). However, in Preston and Gabrieli (2008) the critical comparisons between recognized and unrecognized contexts were made on the basis of only a single recognition trial (i.e., a repeated display was classified as “explicit” if it was correctly recognized as having been seen before on only one recognition trial). Although it is somewhat arbitrary to determine when a repeated context is explicit, it is questionable whether the criteria used in this study really index explicit display knowledge. Further, in this study search and recognition performance were obtained in different sessions (and environments), which may prevent a “clean” comparison of the dependent variables. Another study found that neural activity, in a variety of MTL regions (such as the HC or perirhinal cortex), was greater for “explicit” than “implicit” observers (Westerberg et al., 2011). Note that “explicit” observers in this study were given the opportunity to explicitly learn the repeated contexts prior to search experiment. In contrast, “implicit” observers encountered the repeated displays only at the start of the search task. However, in this study, also observers in the implicit group showed above-chance recognition of repeated displays. This makes the implicit-explicit manipulation questionable. Further, and related to the former point, rather than using T and L stimuli this study used realistic scenes, which makes it problematic to link its findings to previous investigations of the contextual cueing effect. For example, we do know since, for instance, Brockmole and Henderson (2006) that memory for real scenes in visual search is explicit. Applied to Westerberg et al. (2011) this could mean that this study investigated explicit learning—in contrast to implicit learning in “standard” contextual cueing tasks.

A second aim of the present study was to examine at which processing stage MTL structures may play a role in contextual cueing. Prior studies of implicit learning using the serial RT task (e.g., Nissen and Bullemer, 1987) suggest a distinction between the learning of repeated information (i.e., acquisition of memory traces) and the expression of learned information (i.e., retrieval of memory traces; e.g., Frensch et al., 1998). More recently, the distinction between learning and the expression of learning has also been demonstrated for visual search (Jiang and Leung, 2005; Manginelli et al., in preparation). In more detail, in Manginelli et al. (in preparation), the search experiment was divided into a learning phase (trials 1–360) and a test phase (trials 361–480). Importantly, the search task was combined with a secondary spatial working memory (sWM) task that was applied in either the training or the test phase. Note that the sWM task was intended to take away WM resources from the search task and, thus, the learning of repeated distractor contexts. The results showed reliable contextual cueing when the sWM task was administered in the learning phase (see also Vickery et al., 2010), but not when administered in the test phase. Manginelli et al. (in preparation), took this to mean that the expression of learned target-distractor associations depend on sWM. Thus, and given that both HC and PHC support retrieval not only from long-term, but also from WM (Cabeza et al., 2002; Öztekin et al., 2009), one could also assume that these structures are more strongly involved in the expression, rather than the learning, of target-distractor configurations in visual search. Previous imaging studies (e.g., Greene et al., 2007; Preston and Gabrieli, 2008) did not draw a distinction between the learning of repeated target-distractor configurations and their expression in visual search (as well as that between explicit and implicit displays)^1^.

Given this, in the present study, fMRI was administered prior to and after the learning of repeated search displays. The purpose of the fMRI session conducted prior to the learning session was to provide measures as to whether MTL activity differentiates explicit- from implicit-repeated displays at an early learning stage. In contrast, BOLD responses obtained after the learning session should be diagnostic as to whether MTL activity differs between the two types of display at a later stage, when learned contexts may be expressed in search facilitation. The prediction deriving from explicit MTL processes (learning, retrieval) was that of differential activity in MTL structures (HC, aPHC, and pPHC) for explicit compared to baseline (i.e., non-repeated) displays. Further, and in agreement with the “expression-of-learning-hypothesis” (Manginelli et al., in preparation), any activation differences between explicit and baseline displays should become manifest especially on late experimental trials (i.e., fMRI-after-learning group), that is, after sufficient practice on the experimental task. Of course, it is also possible that MTL structures are involved in implicit as well as explicit learning of distractor contexts. If so, MTL activity should differ both for explicit and for implicit relative to baseline displays. Note that the two hypotheses are neutral with regard to the direction of the change—as prior studies have shown evidence for both increased and, respectively, decreased activity of repeated compared to baseline displays (Greene et al., 2007; Preston and Gabrieli, 2008).

## Experiment

### Method

#### Participants

A total of 42 observers (15 female; mean age: 23.7 years) took part in the experiment. Half of them were randomly assigned to one of the two groups (“fMRI-before-learning” vs. “fMRI-after-learning” groups). All participants reported normal or corrected-to-normal vision. They were naïve about the intentions of the study and gave informed consent prior to their participation. They were paid at a rate of €18.00 for both sessions. One participant was excluded due to abnormal ventricle size, another due to a disruption in the experimental procedure, leaving 20 observers in each group.

#### Apparatus

The learning session was conducted in a dimly lighted laboratory, to minimize reflections on the monitor. Stimulus presentation and RT measurement were controlled by a standard PC (a 3.8 GHz pentium). Stimuli were presented on a 22-inch CRT color monitor (refresh rate: 100 Hz), with a resolution of 1280 × 1024 pixels. The experimental control software was purpose-written in C++. Observers viewed the monitor from a distance of approximately 60 cm, maintained by the use of a chin rest. During the fMRI session, stimuli were presented via a DLA-G150CL video projector (JVC Ltd.) with a resolution of 1280 × 1024 pixels (refresh rate: 75 Hz). The stimuli were projected on a rear projection screen placed at the head end of the patient table. The screen was visible to observers through a mirror of high optical quality.

#### Stimuli

**Figure 1:**
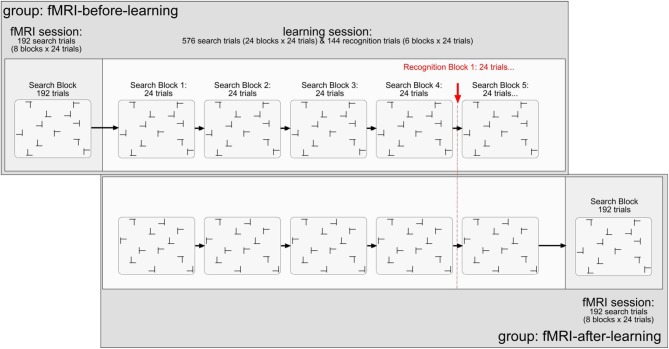
**Illustration of the stimuli and design used in the present experiment.** Explanations are given in the text.

The stimuli were black T's and L's (0.5 cd/m^2^; learning session: 1.35° × 1.35°; scanning session: 1.17° × 1.17°; note that the stimulus displays were smaller in the scanning than in the learning session by a constant factor of 0.86). Targets were T's rotated (in clockwise direction) by 90° or 270° from the vertical, and distractors L's rotated by 0°, 90°, 180°, or 270° rotated L's. Figure 1 illustrates the stimuli and the design of the experiment. Each trial started with the presentation of a black fixation cross in the middle of the monitor for 500 ms (0.5 cd/m^2^; learning session: 0.75°; scanning session: 0.65°). After a blank interval of 200 ms, the search items appeared. The L distractors had a relatively large offset (learning session: 0.28°; scanning session: 0.25°) at their line junction, increasing their similarity with the target and making search relatively difficult (Jiang and Chun, 2001). Each search display consisted of 12 stimuli, which were randomly scattered across the cells of an invisible 8 × 6 matrix (matrix size, learning session: 21.27° × 14.93°; scanning session: 18.38° × 13.76°). The placement of the stimuli within the display matrix was slightly jittered, with the horizontal and vertical distances between adjacent stimuli varying randomly between 1.56° and 1.91° (learning session; scanning session: 1.35° and 1.65°). Observers' task was to detect the T target letter (present on each trial) and to discriminate its orientation (left vs. right) by pressing the corresponding key on the computer keyboard (“Z” or, respectively, “N” key). Observers were asked to respond as fast as and as accurately as possible. Error feedback was provided visually by the presentation of the word “Fehler” (German word for “Error”), in black letters, in the screen center. The intertrial interval was constant at 1000 ms (2000 ms following error trials). For the scanning session, no error feedback was provided and inter-trial intervals were adjusted variably depending on observers' individual trial RT. In doing so, each trial and the subsequent inter stimulus interval added up to 7000 ms. Responses were recorded via an fMRI-compatible infrared-controlled response box (LUMItouch, Photon Control Inc., Burnaby, Canada). Observers were instructed to respond to the orientation of the target (left- vs. right-oriented) by pressing the corresponding buttons with their left and right index fingers, respectively.

#### Design and procedure

All observers performed two experimental sessions: scanning and learning. For half of the observers, the scanning preceded the learning session (and vice versa for the other half). The scanning session comprised of 192 search trials (divided into 8 blocks of 24 search trials each; cf. Chun and Jiang, 1998), performed in a single block (and functional scan). A repeated display was presented on half the trials (a non-repeated on the other half). The dependent variable was the change of the BOLD signal in percent. The independent variables were: (1) memory type (explicit-repeated vs. implicit-repeated display; note that a given value represents the difference in activation between an explicit- or implicit-repeated and non-repeated display, respectively; further, a repeated display was classified as “explicit” if its associated hit rate, obtained on recognition trials of the learning session, was larger than 0.75—see also the Results section^2^); (2) region of interest (ROI; HC vs. aPHC vs. pPHC); (3) brain hemisphere (left vs. right); and (4) experimental group (fMRI-before-learning vs. fMRI-after-learning groups).

The learning session consisted of 576 search trials (divided in 24 blocks of 24 search trials each), in addition to 144 recognition trials (i.e., 6 blocks × 24 recognition trials; 50% repeated displays; 50% newly composed displays). For the learning task, the dependent variable was RT. The independent variables were: (1) display type (repeated display vs. non-repeated display); (2) epoch (1–6; note that four search blocks were aggregated in one epoch to obtain a reasonable estimate of search performance); and (3) group (fMRI-before-learning vs. fMRI-after-learning groups). For the repeated condition, there were 12 randomly arranged target-distractor layouts, generated at the beginning of the search task, which were repeated on randomly selected trials throughout the learning session. Non-repeated target-distractor arrangements were generated on-line on a given experimental trial. In half the trials, a repeated arrangement was presented, and a non-repeated arrangement in the other half. Note that within a given repeated display the orientation of the T target letter could randomly vary across trials. To equate target location repetition effects between repeated and non-repeated displays, the target appeared equally often at each of 24 possible locations throughout the experiment: 12 locations were used for repeated and 12 (different) locations for non-repeated displays. Importantly, observers encountered the very same repeated target-distractor displays in the learning and fMRI sessions (with these displays, of course, being different between observers).

After each forth block of search trials (in the learning session), observers performed a recognition test; this was designed to examine whether they could explicitly discern repeated from non-repeated displays. On each recognition trial, observers were to indicate whether they believed they had seen a given display already in the search task by pressing corresponding key, in an unspeeded manner, on the computer keyboard (“Z” key: “Yes, I have seen/I believe to have seen this display already in the search task”; “N” key: “No I haven't seen/I believe not to have seen this display in the search task”). Thus, a recognition trial again contained the 12 search stimuli (1 target, 11 distractors), except that observers had not to search for the target, but judge the display as seen before or not. Because search and recognition trials were presented concurrently in the learning session, participants were informed about the specific task to be performed (search, recognition) at the beginning of the respective block. In addition, on each recognition trial, the message “Rekognitionsaufgabe” (German word for “recognition task”) was presented at the top of each (to-be-judged) display, to mark these displays unambiguously as recognition displays. Note that for both groups (fMRI-after, fMRI-before), observers only received the instruction to perform the visual search task. This means also that they were not informed about the insertion of the recognition trials, offering no incentive to them to deliberately learn repeated displays.

The learning and fMRI sessions lasted approximately 1 hour each. They were separated by at least 1 day, but not more than 3 days. Prior to the experiment, observers practiced the experimental task in a total of 24 trials (data not recorded).

#### fMRI methods

Magnetic resonance images were acquired with a 3 Tesla Siemens MAGNETOM Trio Scanner equipped with an eight-channel head coil, at the Center for Advanced Imaging, Magdeburg. For each observer, 685 T2^*^-weighted echo-planar-images were acquired. The 32 slices were transversally oriented parallel to the anterior-posterior commissural plane and covered the whole brain (except for a small dorsal area around the central sulcus). Isotropic resolution of the 64 × 64 voxel matrix was 3 mm, with an interslice gap of 10%. Repetition time was 2000 ms (*TE* = 30 ms, *FA* = 80°). Slices were acquired in ascending interleaved order. Recording time for the functional imaging was 22:50 min. For anatomical co-registration, a structural T1-weighted MPRAGE image was acquired before the imaging (192 sagittal slices, 256 × 256 1 mm isotropic voxels, *TR* = 2500 ms, *TE* = 4.77, *FA* = 7°).

#### Pre-processing

MR data were processed using the tools of FSL 4.1 developed by the Oxford Center of fMRI of the Brain (FMRIB; Smith et al., 2004; Woolrich et al., 2009). Time series of the fMRI data were aligned to the first recorded image to adjust for head motion, by using the routine MCFLIRT which minimizes the deviations by a normalized correlation cost function (Jenkinson et al., 2002). Rigid body transformations of translation and rotation in all axis (6 degrees-of-freedom) were permitted. Slice time correction was applied. After segregation and extraction of the brain from surrounding tissue using BET (Smith, 2002), functional data were smoothed applying a Gaussian kernel with a full-width-to-half-maximum of 5 mm. The time series was high-pass filtered in the temporal domain with a frequency cutoff of 60 s.

#### fMRI analysis

Three regressors of interest were introduced into the general linear model (GLM). (1) The onset of explicit-repeated displays; (2) the onset of implicit-repeated displays; and (3) the onset of non-repeated displays. Duration of the events was set to zero. The onset function was convolved with a gamma function (standard deviation: 3 s, mean lag: 6 s), the default setting of FSL. The first temporal derivative was added as regressor of “no interest” in order to capture individual differences in the temporal dynamics of the hemodynamic response function. Additionally, the head motion parameters were included as six “no-interest” regressors. A further “no-interest” regressor of the onsets of trials divided by the z-scored trial RTs was introduced in order to account for RT differences due to the variable inter-stimulus intervals. Accordingly, duration of the modulator events was set to zero. The final model was filtered applying the same high pass-filter that was used earlier on the functional data. Model estimation was carried out using FILM (FMRIB's Improved Linear Model) after pre-whitening of the GLM data, minimizing time series autocorrelation, and increasing validity and efficiency of statistics (Woolrich et al., 2001). Functional images were standardized by a 7-degrees-of-freedom co-registration to the structural image limiting transformation to rotation and translation in all axis, and global scaling and a 12-degrees-of-freedom co-registration of the structural image to the MNI (Montreal Neurological Institute) T1-template (ICBM152) allowing transformation of rotation, translation, scaling, and shearing in all axes. Contrast maps were brought into the MNI standard space by using FLIRT (FMRIB's Linear Image Registration Tool; Jenkinson et al., 2002). To investigate signal changes in the ROIs (HC, aPHC, pPHC; note that these structures were anatomically determined according to the Harvard–Oxford cortical atlas; the probabilistic ROI were thresholded at 25%; see Table 1), FEATquery (FMRIB's Expert Analysis Tool) contrast parameters were extracted separately for each ROI in the left and the right hemisphere and subsequently converted into percent signal-change.

**Table 1:** **Number of 2-mm isotropic voxels inside the Regions-of-Interest of the medial temporal lobes as defined in Harvard–Oxford cortical atlas; brackets show the mean number of 3-mm isotropic voxels (± standard deviation) after transformation in the subject-native EPI space**.

	**Hippocampus**	**Parahippocampal cortex, anterior division**	**Parahippocampal cortex, posterior division**
Left hemisphere	2840 (453 ± 39)	2969 (447 ± 39)	2264 (365 ± 31)
Right hemisphere	2997 (467 ± 41)	3115 (466 ± 42)	1917 (302 ± 26)

## Results

RTs were analyzed using R (R Development Core Team., 2007). For each experimental condition (display type × epoch), RTs outside the range of ±2.5 standard deviations from the mean were discarded as “outliers” (overall, 2.7% of trials). Error-response trials were also excluded from the analysis (1.1% of trials). Note that a 2 (display type) × 6 (epoch) × 2 (group) mixed-design ANOVA on the error rates revealed no significant effects (all *F*-values <1). The results are presented in the following sections, first for the RT performance, followed by the recognition performance and the theoretically important BOLD effects.

### RT performance

**Figure 2:**
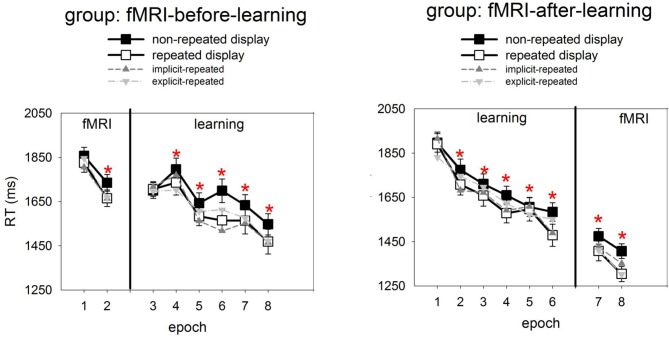
**Mean correct RTs and associated standard errors (in ms) to repeated (white squares) and non-repeated displays (black squares) for the two experimental groups (left panel: fMRI-before-learning group; right panel: fMRI-after-learning group), separately for the fMRI (comprising of 2 epochs) and learning sessions (6 epochs).** Significant RT differences between repeated and non-repeated displays (i.e., contextual cueing) are marked by red asterisks. Also shown are RTs to implicit- and explicit-repeated displays (dark and light grey triangles, respectively).

Figure 2 presents, for the learning session, the RTs to repeated and non-repeated displays as a function of the six experimental epochs, separately for the two groups of observers (fMRI-before- and fMRI-after-learning groups). Also shown are RTs in the two epochs of the fMRI sessions. To examine the effects of repeated target-distractor arrangement on RTs, a mixed-design ANOVA was conducted with the independent variables display type (repeated, non-repeated display; within-subject factor), epoch (1–6; within-subject factor), and group (fMRI-before-learning, fMRI-after-learning groups; between-subject factor). This ANOVA revealed significant main effects of display type, epoch, and a significant epoch × group interaction. No other effects were significant. RTs were faster for repeated relative to non-repeated displays (1637 vs. 1694 ms; 57 ms-effect; *F*_(1, 38)_ = 10.69, *p*< 0.01; main effects of display type). The RT advantage for repeated over non-repeated displays suggests the operation of contextual cueing (e.g., Chun and Jiang, 1998) in the present experiment. Furthermore, RTs became faster as the experiment progressed (first vs. sixth epoch: 1809 vs. 1526 ms; 283 ms-effect; *F*_(5, 190)_ = 33.68, *p* < 0.01; main effect of epoch). The epoch main effect can be attributed to procedural learning—such as improved mapping of a specific target onto a specific response (e.g., Schneider and Shiffrin, 1977). And, as indicated by the significant epoch × group interaction, this effect was less pronounced in the fMRI-before-learning group compared to the fMRI-after-learning group (RT last minus RT first epoch, fMRI-before-learning group: 196 ms-effect; fMRI-after-learning group: 368 ms-effect; *F*_(5, 190)_ = 5.43, *p* < 0.01). Additional *post-hoc* (LSD) tests revealed the interaction to be due mainly to between-group RT differences at the beginning of the experiment (first epoch, fMRI-before-learning group: 1703 ms; fMRI-after-learning group: 1913 ms; *p*< 0.01), rather than the end of the experiment (last epoch, fMRI-before-learning group: 1507 ms; fMRI-after-learning group: 1545 ms; *p* = 0.27). The epoch × group interaction may be taken as evidence that procedural learning already started in the scanning session (in the fMRI-before-learning group), leading to expedited RTs in the subsequent learning session (intended for practicing repeated target-distractor arrangements).

It is worth mentioning that, although the display type × epoch interaction was non-significant [*F*_(2, 200)_ = 1.62, *p* = 0.15], finer-grained (i.e., epoch-wise) analyses of the contextual cueing effect revealed no reliable RT difference between repeated and non-repeated displays for epoch 1 (1803 vs. 1813 ms; one-tailed *t*_(41)_ = 0.10, *p* = 0.46), while there were differences for all subsequent epochs (all *p*-values <0.05).

Analyses of the RT performance in the scanning sessions (mixed-design ANOVA group × epoch × display type; see also Figure 2) also revealed evidence for contextual cueing in both groups (*F*_(1, 38)_ = 10.81, *p* < 0.01; 1551 vs. 1618 ms; 67-ms effect; in addition, the effects of group and epoch were significant). Although the display type main effect suggests that contextual cueing was functional in both groups, further (*post-hoc*) tests were done in order to examine whether configural learning varied across groups and epochs. This analysis revealed that the cueing effect was manifest in epoch 7 and 8 of the fMRI-after-group (epoch 7: 1408 vs. 1474 ms, *p* < 0.05; 66-ms effect; epoch 8: 1304 vs. 1406 ms; *p* < 0.01; 102-ms effect). Further, a reliable contextual cueing effect was also found in epoch 2 of the fMRI-before-group (1665 vs. 1734 ms; *p* < 0.05; 69-ms effect). However, for epoch 1 the effect was non-significant (1825 vs. 1856, *p* = 0.28; 29-ms effect). The latter suggests the contextual cueing developed only later in the fMRI-before-group. Further, the positive finding of RT differences, across sessions, between repeated and non-repeated displays can be taken as evidence that learning of repeated displays lasts for several days and can transfer across consecutive sessions (e.g., Chun and Jiang, 2003) and environments (lab vs. scanner; e.g., Manginelli and Pollmann, 2009).

### Recognition performance

Recognition performance was examined by calculating the hit and false alarm rates for responses to repeated and non-repeated configurations, respectively, across the 144 recognition trials. A “hit” means that a repeated configuration was correctly identified as having been seen before, whereas a “false alarm” means that a non-repeated display was incorrectly judged as having been seen before. Importantly, hit rates were calculated separately for each of the 12 repeated configurations. A repeated configuration was classified as “explicit” if its associated hit rate was larger than the 0.75 correct threshold (cf. Geyer et al., 2010), that is, if a configuration was correctly judged as seen before on at least five out of six recognition trials for this configuration^3^. The analyses of the data then showed that the mean number of “explicit” configurations was 4.2, with a standard deviation of 2.7. Moreover, a comparison of the hit and false alarm rates (with the latter being averaged across all non-repeated displays for a given participant) showed that the hit rates to explicit-repeated displays were reliably higher than the false alarm rates to non-repeated displays [0.87 vs. 0.44; *F*_(2, 38)_ = 219.87, *p* < 0.01; ANOVA group × response type], and this was almost uninfluenced by the scanning regime (fMRI-before-group: 0.88 vs. 0.45; fMRI-after-group: 0.86 vs. 0.44). In contrast, the hit rates to implicit-repeated displays were comparable to the false alarm rates for non-repeated displays [0.47 vs. 0.44; *F*_(1, 38)_ = 1, 62, *p* = 0.21; ANOVA group × response type]. Again, this was not influenced by whether observers participated in the fMRI-before (0.46 vs. 0.45) or fMRI-after group (0.47 vs. 44). This result pattern confirms that observers were aware of some (i.e., 4 ± 2) explicit-repeated displays. Interestingly, RTs in the search task were comparable between the two types of repeated displays [explicit vs. implicit: 1640 and 1634 ms; *F*_(2, 38)_ = 0.02, *p* = 0.90; mixed-design ANOVA group × memory type], and this was observed for both groups (fMRI-before-group: 1768 vs. 1746 ms; *p* = 0.74; fMRI-after-group: 1513 vs. 1523; *p* = 0.89; RTs were collapsed across the learning and scanning sessions). This suggests no processing differences, at the behavioral level, between the two types of displays (see also Figure 2, which shows how RTs to explicit- and implicit-repeated displays developed through the course of the experiment).

### fMRI data

Mean percent-signal-change, obtained by FSLquery ROI analysis, was examined (also using R) in a mixed-design ANOVA with the following factors: ROI (HC, aPHC, pPHC; within-subject factor), hemisphere (left, right; within-subject factor), memory type (explicit-repeated display, implicit-repeated display; within-subject factor), and group (fMRI-before-learning, fMRI-after-learning; between-subject factor). This analysis revealed a main effect of memory type [F_(1,38)_= 5.96, *p* < 0.05], owing to higher signal strength for explicit- compared to implicit-repeated displays (mean percent signal change: 0.013 vs. −0.004). Note that additional tests revealed activation changes associated with the processing of repeated displays to be reliably different from zero only for explicit, but not implicit, displays: explicit-repeated displays, mean percent signal change: 0.013 (one-tailed *t*_(38)_ = 1.70, *p* < 0.05); implicit-repeated displays, mean percent signal change: −0.004 (one-tailed *t*_(38)_ = 0.60, *p* = 0.28). Furthermore, the three-way interaction group × ROI × hemisphere [*F*_(2, 76)_ = 3.70, *p* < 0.05] and the theoretically important four-way interaction memory type × group × ROI × hemisphere [*F*_(2, 76)_ = 3.81, *p* < 0.05] were reliable. No further effects were significant.

**Figure 3:**
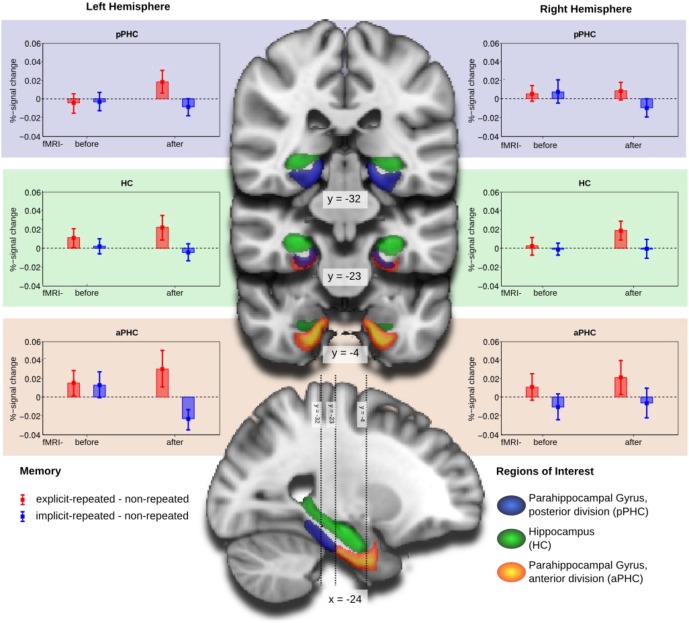
**Group mean and standard error of percent-signal-change of the memory contrasts explicit-repeated minus non-repeated displays (red) and implicit-repeated minus non-repeated displays (blue) for the regions of interest (ROI): parahippocampal gyrus, posterior division (aPHC; upper row), Hippocampus (HC; middle row), and parahippocampal gyrus, anterior division (pPHC; lower row), separately for the left (left column) and right (right column) hemispheres.** Groups are fMRI-before- and fMRI-after-learning. ROIs are projected on coronal slices and one sagittal slice of a standard MNI-template.

The four-way interaction was further explored by separate mixed-design ANOVAs for each of the three ROI's (with memory type, hemisphere, and group as factors). As illustrated in Figure 3, the numerically largest difference between activities associated with the processing of explicit- and, respectively, implicit-repeated displays was evident in the left anterior parahippocampal ROI (aPHC) for observers in the fMRI-after-learning group. This observation was confirmed by a significant memory main effect [*F*_(1, 19)_ = 5.10, *p* < 0.05], in addition to a memory type × hemisphere × group interaction [*F*_(1, 38)_ = 4.83, *p* < 0.05] for the anterior parahippocampal ROI. Additional *post-hoc* tests showed that activity changes were significantly different from zero for both explicit-repeated displays (mean percent signal change: 0.03; one-tailed *t*_(38)_ = 1.66, *p* < 0.05) and implicit-repeated displays (mean percent signal change: −0.02 (one-tailed *t*_(38)_ = 2.31, *p* < 0.05) in the left aPHC.

In contrast, no effects were significant in the posterior parahippocampal ROI or the HC. There was, however, a marginally significant memory type main effect in the hippocampal ROI [*F*_(1, 38)_ = 3.93, *p* = 0.055], reflecting higher activation for explicit- compared to implicit-repeated displays^4^.

### Relations between fMRI and behavioral data

**Figure 4:**
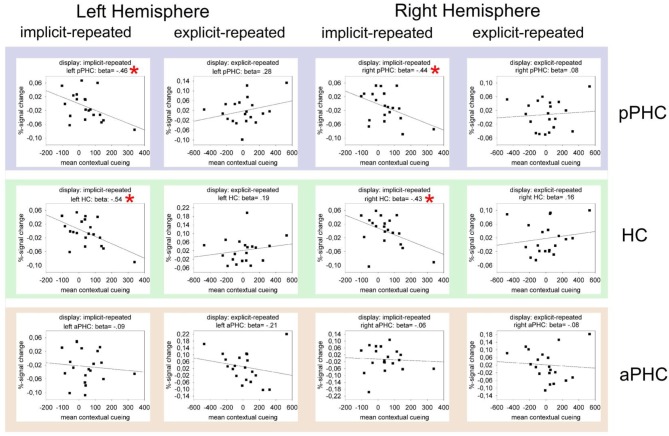
**Mean percent BOLD signal change as a function of contextual cueing (estimated as the RT difference between repeated and non-repeated displays) in the critical ROIs (pPHC, HC, and aPHC; top, middle, and bottom panel, respectively) of the left and right hemisphere.** The data are presented separately for implicit-repeated and explicit-repeated displays and only shown for observers of the fMRI-after-learning group. The black lines represent the best linear regression fit of the data. Also, correlation (i.e., beta) values are given. Significant correlations are marked by a red asterisk.

Figure 4 presents the results of regression analyses carried out to examine the relationship between BOLD activity and behavioral contextual cueing. The data are shown only for the fMRI-after-learning group (as ROI effects were evident only in this group), separately for explicit- and implicit-repeated displays and each of the three ROI's (HC, pPHC, and aPHC) in the left and the right hemisphere. As it can be seen, almost all correlations were positive for explicit displays and negative for implicit displays. In order to examine whether these differences reflect meaningful effects, multiple GLMs were computed with BOLD activity as dependent variable and the magnitude of behavioral contextual cueing as the first (metric) and display type (explicit, implicit) as the second independent variable. In these analyses, differences in (BOLD-activity with behavioral-cueing) correlations between explicit and implicit displays would thus be revealed by an interaction of the two independent variables. Model estimations were conducted separately for each ROI (3 levels) × hemisphere (2 levels) combination. Importantly, *P*-values were adjusted for multiple comparisons using a correction method based on false discovery rates (Benjamini and Yekutieli, 2001). The behavioral cueing × display type interaction reached significance in the left and right HC [left HC: *F*_(1, 17)_ = 6.44, *p*_(corr)_ < 0.05; right HC: *F*_(1, 17)_ = 10.26, *p*_(corr)_ < 0.05], and in the left and right pPHC [left pPHC: *F*_(1, 17)_ = 8.28, *p*_(corr)_< 0.05; right pPHC: *F*_(1, 17)_ = 5.47, *p*_(corr)_ < 0.05]. No evidence of correlation differences between explicit and implicit displays was found for the left and right aPHC (both *p*'s_(corr)_ > 0.40).

In sum, the analyses of the fMRI data revealed processing differences between explicit and implicit displays in all investigated ROI's, with these differences being strongest in the left aPHC for observers in the fMRI-after-learning group—see Figure 3. Moreover, for these observers, BOLD signals were correlated with behavioral contextual cueing associated with explicit (positive correlation) and implicit (negative correlation) displays, with correlation differences being reliable in HC and pPHC of the left and the right hemisphere (see Figure 4). This result pattern indicates that hippocampal and extra-hippocampal areas are involved in the learning of repeated distractor layouts. In particular, extra-hippocampal structures (here: left aPHC) seem to support the processing of both explicit and implicit displays, though, importantly, by means of qualitatively different functional operations, namely, repetition enhancement and, respectively, suppression (discussed below).

## Discussion

### Relations to previous studies: MTL-dependent learning of repeated search displays

The present fMRI experiment re-examined the role of MTL structures, including the HC, in the learning of spatial target-distractor configurations in visual search (Chun and Phelps, 1999; Greene et al., 2007). Prior studies were not fully conclusive as to whether HC or surrounding MTL structures or both contribute to the contextual cueing effect (pro HC: e.g., Chun and Phelps, 1999; Greene et al., 2007; pro MTL: Manns and Squire, 2001; Preston and Gabrieli, 2008), and whether MTL-dependent contextual cueing could be considered as evidence for a unified memory system concerned with the processing of both explicit and implicit information (Greene et al., 2007). The present findings emphasize that before such conclusions can be drawn, it is important to clarify the status of the contextual cueing effect in the first instance—that is: does the effect does really index an implicit (and not an explicit) memory system? In this regard, while previous studies found the effect in the absence of explicit recognition, more recent studies, using more powerful recognition tests, revealed that observers do have explicit knowledge of at least some of the repeated displays (Smyth and Shanks, 2008; Geyer et al., 2010). This suggests awareness in contextual cueing and makes it problematic to link MTL activations associated with the processing of repeated displays to the operation of an implicit memory system (e.g., Greene et al., 2007).

On this background, the present study investigated whether MTL structures were selectively activated by repeated displays of which observers did or, respectively, did not have explicit knowledge. It was found that activation increased after repeated display presentation for explicit displays, but decreased, at least numerically, for implicit displays—in all investigated ROIs: aPHC, pPHC, and HC (main effect of memory type; cf. above). Importantly, activity differences were most pronounced and reliably different from zero only in the left aPHC (significant four-way interaction; see above). This result pattern provides strong evidence for MTL involvement in the processing of explicit and implicit displays. Of note, RT performance was comparable between the two types of display, so that differences in MTL activations cannot be attributed to differences in processing times for these displays.

The increase of BOLD signal strength for explicit vs. non-repeated (baseline) displays is in line with Preston and Gabrieli (2008), who took into account processing differences between “aware” and “non-aware” observers (based on a median-split of their recognition performance). Specifically, Preston and Gabrieli (2008) reported positive correlations, for their aware observers, between explicit memory performance (i.e., corrected hit rate) and activation bilaterally in the HC and PHC. Thus, both Preston and Gabrieli's (2008) and the present study found evidence for a contribution of MTL structures to explicit learning and memory in visual search. The present finding of a BOLD activity decrease for implicit vs. baseline displays is also in line with Greene et al. (2007), who observed exposure to repeated target-distractor configurations to elicit reduced BOLD activity in HC areas. This may also suggest a role of the MTL to the implicit learning of repeated search displays.

In the present study, however, only the (left) parahippocampal gyrus was found to selectively respond to explicit and implicit displays (repetition enhancement vs. suppression). If anything, hippocampal activity was enhanced, rather than suppressed, and this enhancement was observed only for explicit displays (main effect of memory type). As elaborated above, we introduced several manipulations in the design of our study to uncover a possible contribution of the HC to the implicit learning of repeated distractor contexts, including manipulations designed to (1) permit explicit knowledge of display repetitions and RT effects to be assessed concurrently, and (2) test for potential hippocampal involvement in both the learning and the expression of learned distractor contexts. Thus, the simplest remaining conclusion is that extra-hippocampal structures support the formation of explicit and implicit contextual memory representations. This interpretation is not necessarily at odds with the proposal of HC-dependent contextual cueing (Greene et al., 2007). However, it would “reduce” HC-contributions to the processing of explicit, rather than implicit, cueing displays (e.g., Manns and Squire, 2001). Note that this hypothesis presupposes that observers in Greene et al. (2007) did in fact have explicit knowledge of (at least some of the) repeated displays (see, e.g., Smyth and Shanks, 2008). Moreover, given that Greene et al. (2007) found repetition suppression for repeated displays, further studies will be required to clarify the functional operations (repetition enhancement/suppression) of HC that support the learning of (explicit) search displays.

### MTL contributes to retrieval, not acquisition, of memory for repeated distractor contexts

A second aspect of the present study was to clarify whether MTL structures contribute to the learning of target-distractor configurations and/or the expression of learned configurations. Note that, in the present terms, the notion of “expression of learning” refers to multiple memory processes—such as the retrieval of learned target-distractor configurations from long-term memory and/or the maintenance of these configurations in WM in order to aid visual search (see Manginelli et al., in preparation). To examine this question, observers were assigned to an fMRI-before-learning group (performing some 150 “fMRI trials” followed by some 600 “learning trials”) or, respectively, an fMRI-after-learning group (performing 600 “learning trials” followed by 150 “fMRI trials”). The results are consistent with the “expression-of-learning-hypothesis,” in that activation differences between explicit- and implicit-repeated displays were most pronounced after learning and particularly in the left aPHC. But note that, and although we did not find reliable activation differences between repeated and non-repeated displays in the fMRI-before-group, this does not necessarily mean that “early” learning effects are absent in MTL cortex. For example, Preston and Gabrieli (2008), who also investigated the time course of learning in MTL cortex, found that particularly the perirhinal cortex differed repeated from non-repeated displays even after the first two presentations of the former type of displays. In contrast, and in line with the current findings, differential activation in PHC took longer to emerge and was measurable only after about 6–8 presentations of each of the repeated displays. The latter finding also suggests that parahippocampal activation reflects the expression of acquired knowledge.

But there is at least one alternative interpretation of the data, which is related to the current approach of assessing search and recognition performance concurrently. Specifically, observers in the fMRI-before-group were introduced to many repetitions of the search displays without any concurrent recognition trials. In contrast, for the group scanned after the learning session, first exposure to the displays occurred with interleaving recognition trials. This may have lead to the development (and subsequent use) of discrepant learning strategies, in particular: an explicit strategy in the fMRI-after-group, which could explain any differences found between the groups. However, we do consider this alternative explanation unlikely. This reasoning builds up on earlier findings, showing that explicit learning does not affect the contextual cueing effect (Chun and Jiang, 2003). If anything, contextual cueing is reduced under conditions of an explicit learning regime. In more detail, in Chun and Jiang (2003; Experiment 2) observers were informed that half of the displays contained repeated configurations and that they should explicitly encode (i.e., learn) the repeated displays. It was surmised that if explicit learning is a concomitant of contextual cueing, then the cueing effect should be larger in the explicit relative to a baseline condition (i.e., a “standard” cueing experiment, in which observers were not informed about display repetitions). It was found that contextual cueing was reliable in both conditions, but even smaller, numerically, in the explicit condition. Interestingly, post-experimental debriefings revealed that the majority of observers in the explicit condition were still unaware of display repetitions. However, when Chun and Jiang (2003) limited their analysis to a subset of observers, namely those that showed explicit knowledge of repeated displays, the contextual cueing effect was almost absent. In fact, for “aware” observers the cueing effect was only 1 ms. Assuming that also the current approach of assessing visual search and recognition performance simultaneously introduced an explicit strategy (that may have been even more powerful than Chun and Jiang's instructional manipulation), the prediction is that cueing effects should be absent particularly in the fMRI-after-group. However, BOLD activity changes due to contextual cueing were evident only in this group and absent in the other (fMRI-before) group. It is thus unlikely that explicit learning was at play in the fMRI-after-group. As such, both groups were comparable by matters of their respective learning behavior. An alternative view is to acknowledge that observers in the fMRI-after-group may have recognized the repeated displays, but this did not have an effect on their ability to (implicitly) learn these displays, because explicit and implicit learning are distinct phenomena (e.g., Chun and Jiang, 2003). However, explicit recognition performance, in terms of the d prime sensitivity measure (Macmillan and Creelman, 1991), was comparable between the groups (fMRI-before- vs. fMRI-after-group: 0.43 vs. 0.46, *p* = 0.79). This makes the latter thesis unlikely. Instead, it suggests that the concurrent assessment of search and recognition performance did not introduce an explicit learning strategy.

The idea of MTL-dependent memory expression is in line with recent behavioral findings showing that the retrieval, but not the learning, of repeated target-distractor displays in visual search depends on selective attention (e.g., Jiang and Leung, 2005) or the availability of WM resources (Manginelli et al., in preparation; Annac et al., 2011). Concerning the involvement of WM in contextual cueing, eye movement studies show visual search through repeated displays to become more efficient in that fewer saccades and fixations are needed to detect the target (e.g., Manginelli and Pollmann, 2009). Nevertheless, in these studies, typically, the target is not found after a single eye movement; rather, search involves a series of saccades that home in on the target location (e.g., Tseng and Li, 2004). This suggests that detecting a target in a repeated display requires a series of comparisons of the display items with stored memory traces, rather than a single comparison in which the target location is retrieved from long-term memory. Importantly, for this series of retrieval and comparison processes, both long-term memory and WM (buffering currently attended/retrieved items) appear to be necessary. The present data suggest that MTL cortex is involved in these processes, in particular, in the retrieval of learned target-distractor associations.

In more detail, MTL activation to repeated displays was contrasted with activation to non-repeated (“novel”) displays. As all displays are novel at the start of the experiment, no activation differences between the two types of displays were expected at an early experimental phase. And indeed, this pattern was observed for the group of observers who started the experiment in the scanner. Surprisingly though, despite these observers encountering eight repetitions of each of the repeated displays, in addition to showing a behavioral contextual cueing at the end of the scanner session, they showed no difference in BOLD activity between repeated and non-repeated displays. Thus, there was no indication of differential MTL involvement in the learning of repeated displays—importantly irrespective of whether they were later recognized as old with high (i.e., explicit display) or with low reliability (i.e., implicit displays). In contrast, after sufficient repetitions, observers in the fMRI-after-learning group showed a decrease of activation to implicit-repeated relative to non-repeated displays, whereas BOLD responses to explicit-repeated displays were increased compared to responses to non-repeated displays. These differences were significantly different from zero for both explicit- and implicit-repeated displays in the left aPHC. This means that after sufficient repetitions, activation for explicit displays increased above their initial activations, whereas activation for implicit displays decreased across repetitions in the left aPHC.

### Anatomically linked, but functionally distinct explicit and implicit memory processes in MTL

The above result also suggests that the processing of explicit and implicit displays was mediated by the same brain region, namely: the left aPHC, but that the neuronal mechanisms were qualitatively different. In case of explicit displays, the increase of activation across repetitions may indicate enhanced “engagement” of the left aPHC in response to these displays, which may expedite memory retrieval processes and/or the allocation of attention. In contrast, repetition suppression in response to the presentation of implicit displays may reflect neuronal changes related to increased processing efficiency when identical distractor contexts are repeatedly presented (cf. Desimone, 1996; Krekelberg et al., 2006; ^5^). Again, this may facilitate memory retrieval and/or attentional processes.

Similar dissociations between explicit and implicit memory have been noted before, for instance, repetition suppression in the fusiform gyrus under conditions of implicit processing of faces (Henson et al., 2002). In good agreement with the present findings, Weis et al. (2004) reported a dissociation between increased MTL activation for successful explicit context retrieval and decreased MTL activation for item recollection (in the absence of contextual recollection). This dissociation was interpreted within a dual-process framework of activation increase reflecting episodic retrieval, while item (old-new) recognition in the absence of successful context retrieval may be served by familiarity, accompanied by repetition suppression (cf. Henson et al., 2003). However, the present results differ from these previous reports in that we found either repetition suppression or enhancement on a trial-by-trial basis within the same task under identical instructions. This thus emphasizes the importance of an accurate assessment of the implicit vs. explicit nature of processing at the level of individual displays.

In summary, the present fMRI experiment shows that (1) RT gains in repeated visual search (i.e., contextual cueing) are associated with activation changes in left aPHC and that (2) left aPHC responds differentially dependent on whether observers do or do not have explicit knowledge of repeated displays. Interestingly, these processing differences were obtained only when observers were given the opportunity to extensively practice the task, indicating that the left aPHC contributes to the expression of explicitly learned target-distractor configurations, rather than to their learning per se. Furthermore, the data are compatible with the novel view of MTL-dependent explicit and implicit memory processes (e.g., Henke, 2010). However, while repetition effects in visual search are most likely being supported by a single memory system, they are actually expressed by qualitatively different functional operations: explicit displays may induce some form of top–down influence from left aPHC on memory retrieval and/or attentional processes. Implicit displays, by contrast, may lead to bottom–up changes in the efficiency of memory and/or attention functions supported by the left aPHC.

Overall, the current study clearly argues for the need to take into account observers' knowledge about display repetitions (see, e.g., Smyth and Shanks, 2008; present study) in order to derive conclusions about the brain areas underlying explicit and implicit memory effects in visual search.

### Conflict of interest statement

The authors declare that the research was conducted in the absence of any commercial or financial relationships that could be construed as a potential conflict of interest.

## Footnotes

^1^Note that in the present experiment, we do not attempt to draw a strict distinction between learning and retrieval processes, which is notoriously difficult (e.g., Rugg and Wilding, 2000).

^2^Given that explicit displays were determined on the basis of the recognition data obtained in the learning session, one would have to assume, particularly for observers in the fMRI-before-group, that displays recognized in the learning session were also recognized in the scanning session.

^3^In a control analysis, explicit displays were also determined on the basis of both hit and false alarm rates, in order to account for response factors (such as a tendency to say “yes”). In this analysis, a repeated display was classified as “explicit” if its associated d prime value (i.e., d prime = *z*[hit] minus *z*[false alarm]; cf. Macmillan and Creelman, 1991) was equal to or greater than 1, indicating at least 75% correct discrimination performance. The results suggest that the discernment of explicit displays on the basis of the hit rates only is a valid approach and that response factors are unlikely to have influenced recognition performance. In particular, the coincidence rate between individual repeated displays determined as explicit on the basis of hit rates and d prime values was high: 83,7%. And this was relatively constant across both groups (fMRI-before: 82,1%; fMRI-after: 85,2%). This means that a given individual display that was detected as explicit (implicit) on the basis of its associated hit rate was also detected as explicit (implicit) by means of its associated d prime value with an accuracy of approx. 84%.

^4^We also performed a whole brain analysis in order to test whether additional cortical areas outside the critical MTL structures contribute to explicit and implicit learning in visual search. This analysis revealed no significant difference between explicit- and implicit-repeated displays (cluster forming threshold of *z*-value > 2.3 and a corrected cluster significance threshold of *p* < 0.05), neither in the fMRI-before nor fMRI-after group. Also for the group × display type interaction no cluster reached significance.

^5^An alternative proposal is that the reduced response to implicit displays over the course of learning may reflect the reduction of the well-known response to novel stimuli (Grunwald et al., 1998).
